# Orthostatic Challenge Causes Distinctive Symptomatic, Hemodynamic and Cognitive Responses in Long COVID and Myalgic Encephalomyelitis/Chronic Fatigue Syndrome

**DOI:** 10.3389/fmed.2022.917019

**Published:** 2022-06-23

**Authors:** Suzanne D. Vernon, Sherlyn Funk, Lucinda Bateman, Gregory J. Stoddard, Sarah Hammer, Karen Sullivan, Jennifer Bell, Saeed Abbaszadeh, W. Ian Lipkin, Anthony L. Komaroff

**Affiliations:** ^1^The Bateman Horne Center of Excellence, Salt Lake City, UT, United States; ^2^Department of Family & Preventive Medicine, University of Utah School of Medicine, Salt Lake City, UT, United States; ^3^Center for Solutions for ME/CFS, Center for Infection and Immunity, Mailman School of Public Health, Columbia University, New York, NY, United States; ^4^Division of General Medicine, Department of Medicine, Brigham and Women's Hospital, Harvard Medical School, Boston, MA, United States

**Keywords:** long COVID, post-acute sequelae of SARS-CoV-2 infection (PASC), myalgic encephalomyelitis, chronic fatigue syndrome (CFS), orthostatic intolerance (OI), postural orthostatic tachycardia syndrome (POTS), autonomic nervous system dysfunction

## Abstract

**Background:**

Some patients with acute COVID-19 are left with persistent, debilitating fatigue, cognitive impairment (“brain fog”), orthostatic intolerance (OI) and other symptoms (“Long COVID”). Many of the symptoms are like those of other post-infectious fatigue syndromes and may meet criteria for myalgic encephalomyelitis/chronic fatigue syndrome (ME/CFS). Common diagnostic laboratory tests are often unrevealing.

**Methods:**

We evaluated whether a simple, standardized, office-based test of OI, the 10-min NASA Lean Test (NLT), would aggravate symptoms and produce objective hemodynamic and cognitive abnormalities, the latter being evaluated by a simple smart phone-based app.

**Participants:**

People with Long COVID (*N* = 42), ME/CFS (*N* = 26) and healthy control subjects (*N* = 20) were studied just before, during, immediately after, 2 and 7 days following completion of the NLT.

**Results:**

The NLT provoked a worsening of symptoms in the two patient groups but not in healthy control subjects, and the severity of all symptoms was similar and significantly worse in the two patient groups than in the control subjects (*p* < 0.001). In the two patient groups, particularly those with Long COVID, the NLT provoked a marked and progressive narrowing in the pulse pressure. All three cognitive measures of reaction time worsened in the two patient groups immediately following the NLT, compared to the healthy control subjects, particularly in the Procedural Reaction Time (*p* < 0.01).

**Conclusions:**

A test of orthostatic stress easily performed in an office setting reveals different symptomatic, hemodynamic and cognitive abnormalities in people with Long COVID and ME/CFS, compared to healthy control subjects. Thus, an orthostatic challenge easily performed in an office setting, and the use of a smart phone app to assess cognition, can provide objective confirmation of the orthostatic intolerance and brain fog reported by patients with Long COVID and ME/CFS.

## Introduction

Following acute COVID-19, some patients develop a group of debilitating symptoms: fatigue, orthostatic symptoms, difficulty with attention and concentration (often called “brain fog”), myalgias and disrupted sleep (1–8). The syndrome is called “post-acute COVID syndrome”, “long hauler COVID”, or “Long COVID.” We use the last term here. No formal case definition has yet been adopted. The frequency with which this syndrome occurs following acute COVID-19 is unclear, with estimates ranging from 2 to 40% of cases (8, 9). Although individuals with more severe acute COVID-19 appear more likely than those with less severe acute COVID-19 to develop the syndrome, Long COVID still can occur in people who were mildly symptomatic or asymptomatic at the time of acute infection with SARS-CoV-2 (8, 10, 11).

Many people with Long COVID have remained ill for months. Given that the agent implicated in this syndrome, SARS-CoV-2, has only produced human illness since late 2019, it remains unclear how long this illness will last. One systematic review found that fatigue still was present 60 days after confirmed acute COVID-19 in 40% of people (9). Many people with the illness are sufficiently hobbled by symptoms that they cannot return to their pre-COVID level of function—at home or at work. Long COVID is a term used to capture the condition of people who have prolonged multisystem illness after acute infection with SARS-CoV-2, one of several different post-acute sequelae of acute COVID-19 (PASC).

The underlying pathogenesis of Long COVID is being intensely investigated. Chronic injury to the lungs, heart, brain, kidneys, the immune system and other organs may be responsible for some of the symptoms.

More severe forms of Long COVID can be clinically similar to the illness myalgic encephalomyelitis /chronic fatigue syndrome (ME/CFS) (11–13), as well as to the post-infectious fatigue syndromes which follow a variety of viral, bacterial and protozoal infections (1). It has been estimated that 13%−46% of people with lingering symptoms following acute COVID-19 meet criteria for ME/CFS (14, 15).

In many people with ME/CFS, the chronic illness starts with an “infectious-like” acute illness characterized by respiratory and gastrointestinal symptoms, myalgias, lymphadenopathy, fevers, fatigue and other symptoms. People with ME/CFS often remain ill for many years, with at least a quarter being periodically house- or bed-bound (16), and with direct and indirect annual costs in the U.S. of up to $24 billion (17, 18). Community surveys reveal that fewer than 10% of people who have ME/CFS have been diagnosed with the condition (19). A primary characteristic of ME/CFS is post-exertional malaise: a prolonged exacerbation of symptoms after physical, cognitive or orthostatic stress (12).

People with Long COVID and people with ME/CFS often describe orthostatic intolerance (OI)—symptoms during upright posture that are relieved by becoming recumbent—as well as brain fog—difficulty with cognition and attention (12). Typical symptoms of OI include lightheadedness, nausea, cognitive problems, disrupted sleep, headache, palpitations and exercise intolerance (20). In chronic OI, people may experience symptoms that do not occur exclusively during upright posture including nausea, sleep disturbance, neurocognitive deficits and sensitivity to heat or cold (21). While symptoms of OI can occur in otherwise healthy people, this typically is an acute problem triggered by heat, dehydration or emotion that is easily remedied.

When formal testing of autonomic function is performed, some people with OI meet criteria for postural orthostatic tachycardia syndrome (POTS), orthostatic hypotension and neurally-medicated hypotension (22). All of these abnormalities involve dysfunction of the autonomic nervous system (ANS).

Persisting abnormalities of the ANS have been noted in early studies of people with COVID-19 in the months following the acute illness (11, 23–25). ANS abnormalities, particularly involving the cardiovascular system, were also first noted 25 years ago in people with ME/CFS (26, 27), and have been repeatedly confirmed in recent years (28, 29). Indeed, the Institute of Medicine (IOM) identified OI as one of the core diagnostic features of ME/CFS (12).

Clinical testing for OI typically employs head-up tilt table testing with continuous heart rate monitoring performed by specialists after referral from primary care physicians, and requiring additional fees and copayments. In addition, such testing is not always readily available. When a patient presents with symptoms of OI and brain fog, the clinician seeks some *objective* measurement to confirm these symptoms. Objective evidence of autonomic dysfunction may help in the diagnosis of both Long COVID and ME/CFS and may point toward possibly effective therapies.

The purpose of this study was to determine the value to the office-based primary care clinician of simple tests that can easily be used in the office in order to obtain objective evidence of OI and brain fog. Undoubtedly, OI and brain fog can be assessed by more rigorous testing, but for the primary care clinician and the patient this requires the delay and expense of a referral to a specialist.

Specifically, we examined the value of a test easily performed in a primary care practice office: the 10-min National Aeronautics and Space Administration (NASA) Lean Test (NLT). The NLT is a variant of a test used decades ago by NASA investigators to test for orthostatic intolerance (30). It reduces muscular influences on venous return, a major cause of variability in orthostatic testing. Such passive stand testing has been validated as an equivalent or superior measure of orthostatic intolerance as compared to Head-Up Tilt Table tests (31, 32). In contrast to Head-Up Tilt Table tests, which can be performed somewhat differently from one center to another, the NLT is standardized as to body position and timing of measurements.

The NLT previously has been studied in people with ME/CFS and healthy control subjects (29, 33), and found to distinguish the two groups, particularly if measurements are continued for a full 10 min. The duration of the NLT is important because, generally, physicians perform orthostatic assessments for just 1–3 min. Cognition was assessed by the Defense Automated Neurobehavioral Assessment (DANA) Brain Vital app for a patient's smart phone, an easily-used test that rapidly evaluates an individual's reaction time.

## Methods

### Study Subjects

#### People With Long COVID

Between April 2021 and September 2021, 42 people between the age of 18 and 65 years old, with >3 months of persistent symptoms following acute illness due to COVID-19, who had been diagnosed by a clinician with COVID-19 based on clinical presentation and polymerase chain reaction (PCR), antigen or antibody testing, and had diminished activity compared to their pre-illness activity levels were enrolled in this study. Of the 42, 22 were patients at the Bateman Horne Center in Salt Lake City, Utah receiving clinical care for their post-COVID-19 symptoms and 20 were recruited from other practices for participation in the study.

Exclusion criteria included: (1) known medical conditions that might place the patient at risk from, or confound the interpretation of, the NLT; (2) inability to stop beta blocker medication in the 48 h prior to testing; (3) inability to stop using fludrocortisone in the 5 days prior to testing; (4) having a past medical history of HIV, hepatitis B or C, or *Borrelia burgdorferi* infection; (5) not agreeing to limit fluid or sodium intake prior to testing as required by the study protocol; (6) if a women, being pregnant or women <3 months postpartum and lactating; or (7) unable to take the post-test measurements during the week following the NLT, as required by protocol.

#### People With ME/CFS

Between February and November 2020, 26 people meeting the Institute of Medicine criteria for ME/CFS (12) and receiving care for this condition at the Bateman Horne Center were enrolled in this study. At the time of participation in the study, nine people with ME/CFS had been sick for less than 4 years, six had been sick for 5–6 years, and nine had been sick for >10 years. The subjects were 18–65 years old at the time of enrollment. Exclusion criteria were identical to those for people with Long COVID.

#### Healthy Control Subjects

Between February and November 2020, 20 healthy control subjects were enrolled. The healthy control subjects were recruited from the Salt Lake City metropolitan area using advertisements posted on social media and on the clinic webpage, and by phone contact with a healthy volunteer pool from previous studies. The subjects were 18 to 65 years old at the time of enrollment; the same age (within 5 years), gender, race and ethnicity as the people with ME/CFS and described themselves as in good health and able to fulfill their responsibilities at home and at work. Exclusion criteria were the same as for the two patient groups. In addition, possible healthy control subjects were excluded if they had a household contact or close relative with ME/CFS.

#### Functional Level

Participants were asked to select their current activity level as limited (sit-down job, no regular physical activity, 3–4 h of walking or standing per day), active (physically active two or more times a week during leisure time), or demanding (physically demanding occupation, farmer, mail deliverer, stockroom worker, professional athlete, firefighter), using the Patient Activity Questionnaire (courtesy of B. Keller).

### Orthostatic Challenge: NASA Lean Test (NLT)

To assess the manifestations of OI, changes in HR and BP during the 10-min NLT were classified as follows: (1) *Orthostatic hypotension* (OH) was defined as a decrease in SBP of 20 mm Hg or more, or a decrease in DBP of 10 mm Hg or more in the first 3 min standing compared to resting supine values; (2) *Postural orthostatic tachycardia syndrome* (POTS) was defined as an increase of HR >30 bpm at any time during the 10-min NLT.

#### Preparation for the NLT

The evening before the NLT routine evening medications that were not essential for clinical stability were held or reduced to the lowest possible doses based on the participants' comfort and experience. Participants on fludrocortisone were instructed to stop this medication 5 days before testing. Participants taking beta blockers were asked to taper and stop the medications 48 h prior to testing. All participants took any other routine morning medications up until the morning of the NLT; that morning, they brought their morning medications with them to the clinic and took them only after testing was complete.

For ~24 h prior to the orthostatic challenge, participants were instructed to refrain from consumption/use of caffeine, alcohol, tobacco, or the intake of increased amounts salt or fluids (salt and fluid loading). If testing was done within 4 h of arising, participants were able to consume 8–12 ounces of a caffeine-free fluid (e.g., water, juice, milk, Gatorade) up to 60 min prior to the orthostatic challenge. If testing was done >4 h after arising, participants were allowed a light breakfast and 8–12 ounces of a caffeine-free fluid up to 60 min prior before the orthostatic challenge. Participants were instructed to wear loose, comfortable clothing.

#### Conduct of the NLT

The NLT is an orthostatic challenge test that can be performed in a doctor's office, without special equipment (33). It requires an exam table, a pulse oximeter placed on one hand and a blood pressure cuff placed on the opposite arm.

To begin, participants were positioned on the exam table with back support and straight legs (legs not dangling) and instructed to complete the first round of cognitive testing (described below). Then, an uninflated blood pressure (BP) cuff was placed on the participants' arm, following which they rested quietly in a supine position for 10 min. After 10 min, a pulse oximeter was placed on a finger of the arm without the BP cuff. Resting supine measurements of heart rate (HR) using pulse oximeter, and BP were obtained until two consecutive BP readings were within 3 mmHg for both systolic BP and diastolic BP, and 3 bpm for HR.

Then the participant was instructed to slowly sit up, stand and then lean against the wall with heels ~6″ from the wall and shoulder-blades in contact with the wall while BP and HR measurements were taken each minute, ideally for 10 min. Participants were instructed to remain still, relaxed and quiet while NLT measurements were being obtained unless reporting symptomatic responses to the orthostatic testing.

After 10 min of standing, the participants sat on the edge of the exam table or in a chair with feet on the floor or feet dangling and immediately completed the second round of cognitive testing, described below. Following cognitive testing, they rested supine on the exam table until fully recovered.

### Symptom Assessment

Participants verbally rated the severity of six symptoms on a scale of 0–10, with 0 indicating an absence of the symptom and 10 being the worst conceivable severity of the symptom. We could only inquire about a limited number of symptoms because of the brief time (10 min) of the test. The six symptoms chosen were fatigue, brain fog, lightheadedness/ dizziness, nausea, discomfort in the chest and/or with breathing, and pain (located anywhere in the body). This assessment was made at four time points: just prior to the start of the NLT (baseline), in the ninth minute of the NLT, immediately after the NLT, 2 days later and 7 days later.

### Hemodynamic Measurements

Systolic BP (SBP), diastolic BP (DBP) and heart rate (HR) were measured just prior to the start of the NLT and each minute during the NLT, and immediately after the test was completed. From these raw values we calculated the Pulse Pressure (PP), which is the difference between systolic and diastolic blood pressures (PP = SBP – DBP). We then determined whether the ratio of PP/SBP × 100 indicated an abnormally narrowed PP: a value of <25% of is considered abnormally narrowed (34).

### Cognitive Testing

Prior to arriving at BHC for the orthostatic challenge, participants downloaded to their smart phones a point-of-care cognitive testing app: the Defense Automated Neurobehavioral Assessment (DANA) Brain Vital app. Cognitive testing was performed on four occasions: just before the NLT, immediately after the NLT, 2 days later and 7 days later.

DANA Brain Vital is a test that includes three reaction time measurements: procedural reaction time (PRT), simple reaction time (SRT), and sustained attention or Go-No-Go (GNG) (35). The test results are measurements of cognitive efficiency and are calculated as accuracy × speed × 60,000. The PRT measures reaction time in making a choice between options. The user differentiates between two sets of characters: a 2, 3, 4, or 5 appears on the screen and the user taps one of two buttons [2 or 3] or [4 or 5]. The PRT is a more complex reaction time task than the simple reaction time (SRT) in which the user taps an orange target symbol as soon as it appears. SRT starts with five practice trials, followed by 40 testing trials. GNG is a forced choice measure of reaction time: (1) a building with several windows is presented on the screen; (2) either a gray “foe” or a green “friend” appears in a window; and (3) the user taps only when a “foe” appears. GNG has five practice trials and 30 testing trials.

### Statistical Analysis

Basic demographic data of the three groups was compared in Excel using the *t*-test or ANOVA for continuous variables and the chi-square or Fisher exact tests for categorical variables, with a *p* value of <0.05 accepted as statistically significant.

The R system for statistical computing was used to conduct the following analyses (36). To evaluate the impact of orthostatic challenge on hemodynamics and cognition in the three groups, we employed linear regression models. We used a mixed effects linear regression model to model the outcomes at the average and maximum orthostatic stress time points. A linear relationship was assumed to be a sufficiently good approximation of the underlying actual shape of the means of the 10 repeated hemodynamic measurements. Using post-fit marginal estimation, the mean SBP, DBP, and HR at 5.5 and 10 min was estimated, which are simply points on the regression lines at those values of time (the *X*-axis variable). Because 4/26 people with ME/CFS, 7/42 with Long COVID, 1/20 healthy control subjects (*p* = 0.17, Fisher exact test) did not complete the NLT, missing BP values were replaced by the last previous non-missing value as the missing data imputation approach. When subjects could not complete the full 10-min NLT, it was because of symptoms of orthostatic intolerance: presyncope, lightheadedness, weakness in their legs, dizziness, blurry vision, nausea or the need to vomit, chest heaviness, feeling cold or fainting.

Analysis of covariance (ANCOVA) was used to control for any differences between the three groups in baseline hemodynamic measurements (e.g., SBP, DBP, and HR) and cognitive measurements (e.g., SRT, PRT, and GNG) by including the baseline outcome as a covariate in the model. The models included time as a continuous predictor and group, group × time interaction as fixed effects. All pre-test cognitive efficiency values were normalized to 0. The mean of the three baseline readings was included as a fixed effect covariate in ANCOVA models. Both a random intercept and random slope across time was specified for each participant, with participant as a random effect. Sex and age dropped out of all models as nonsignificant, using backwards variable selection.

A linear mixed model was used for symptom data collected during and after the 10-min NLT. Adjusted means were calculated, from the linear mixed model, to compare the ME/CFS, Long COVID and HC group results. The marginal effects command was used to show the differences between the adjusted mean values along with a 95% CI and *p*-value. Symptom data on Day 2 post-test was not completed in three of the 88 study subjects, and symptom data on Day 7 post-test was not completed in two of the 88 study subjects.

## Results

### Study Subjects

As shown in Table 1, the ME/CFS patients and healthy control subjects were similar in age; Long COVID patients were somewhat older (*p* < 0.02). The distribution of sex, race, ethnicity, and BMI between the three groups was similar. All Long COVID patients and all but one ME/CFS patients indicated that they had limited physical activity. For the Long COVID patients, the period of limited physical activity had lasted, on average, for 6 months; for the ME/CFS patients, it had lasted, on average, 7 years.

**Table 1:** Study participant characteristics.

	**Long COVID (*N* = 42)**	**ME/CFS** **(*N* = 26)**	**Healthy controls (*N* = 20)**
Age in years ± SD	43.5 ± 9*	37 ± 12	35 ± 14*
Sex			
Male	4 (10%)	8 (31%)	6 (30%)
Female	38 (90%)	18 (69%)	14 (70%)
Race			
Asian	0 (0%)	2 (8%)	2 (10%)
White	41 (98%)	24 (92%)	18 (90%)
Multiracial	1 (2%)	0	0
Ethnicity			
Hispanic	6 (14%)	2 (8%)	1 (5%)
Not Hispanic	36 (86%)	24 (92%)	19 (95%)
BMI ± SD	27.6 ± 5	26 ± 7	26 ± 3
Activity level			
Limited	42 (100%)	25 (96%)	1 (5%)
Active	0**	1 (4%)**	19 (95%)**
Demanding	0	0	0
Years at current activity level ± SD	0.6 (± 0.2)	7 ± 6	

* *Difference between the three groups significant at p < 0.02.*

** *Difference between the three groups significant at p < 0.001*.

### Symptoms

**Figure 1:**
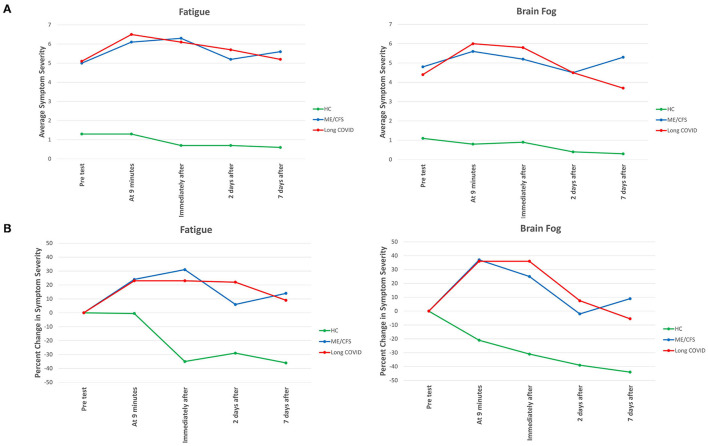
Severity of fatigue and brain fog at different time points before, during and after NASA Lean Test in Long COVID, ME/CFS and healthy control subjects. In **(A)**, the *absolute severity* level at each time point is displayed. In **(B)**, the *percent change* from just before the Test at each of the different time points is displayed. In **(A)**, all differences between the two patient groups and the healthy control subjects are statistically significant (*p* < 0.001). In **(B)**, the same is true for each time point during and following the Test.

For two core symptoms, fatigue and brain fog, Figure 1A displays the severity of two symptoms—fatigue and brain fog—at baseline and at each of the four subsequent time points; Figure 1B displays the change from baseline in symptom severity at each time point. The patterns for the other four symptoms were similar (data not shown).

As was expected, the severity of each symptom was significantly greater at baseline for those with illness. For each of the six symptoms, at each of the five time points, the differences in severity between the people with Long COVID vs. healthy control subjects, and between the people with ME/CFS and healthy control subjects, was highly significant (*p* < 0.001). In contrast, there was no significant difference in the severity of any symptom between people with Long COVID and people with ME/CFS at any time point.

Not only was symptom severity greater in the two patient groups than the healthy control subjects, but a different *pattern* of symptom severity was also apparent. As seen in Figure 1B, in the Long COVID and ME/CFS patients, compared to baseline (pre-test) levels, symptom severity rose considerably during and following the NLT, whereas in the healthy control subjects symptom severity typically dropped immediately after ending the NLT.

In the Long COVID patients, the severity of several symptoms (brain fog, dizziness, nausea, chest pain, pain anywhere) had returned to baseline levels by Day 7; in the ME/CFS patients, only nausea had returned to baseline levels.

Several symptoms improved by Day 2 post-test, but then flared again and worsened by Day 7: in the Long COVID patients, that occurred with chest pain; in the ME/CFS patients that occurred with brain fog, chest pain, and pain anywhere.

### Hemodynamic Measurements

As shown in Table 1, one Long COVID patient, and no ME/CFS or HC, had OH. POTS was significantly more common in ME/CFS patients (12/26, 46%) than in HCs (5/26, 19%, *p* = 0.04). POTS was not significantly more common in Long COVID (12/42, 28%) compared to ME/CFS (*p* = 0.14) or HCs (*p* = 0.4). ME/CFS patients were about three times more likely to experience presyncope or syncope than HC, and Long COVID were about four times more likely but the incidence percentages were not statistically different from HCs (Fisher exact test *p* = 0.21).

As shown in Table 2, SBP was not significantly different between the three groups, at any of the three time points. Heart rate was somewhat higher at completion of the NLT in both Long COVID and ME/CFS patients, compared to healthy control subjects.

**Table 2:** Hemodynamic measurements in each of the three groups at three time points of the NASA Lean Test, and percentage of people meeting orthostatic intolerance criteria.

	**Long COVID (*N* = 42)**	**ME/CFS** **(*N* = 26)**	**Healthy controls (*N* = 20)**
Heart rate			
Pre-test	68.4	68.9	63.7
At 5.5 min	92.8	93.7	88.4
At 10 min	**96.4 (*****p*** **=** **0.05)**	**98.1 (*****p*** **=** **0.02)**	90.4
Systolic BP			
Pre-test	114.2	116.6	113.7
At 5.5 min	114.5	117.2	114.5
At 10 min	113	116	113
Diastolic BP			
Pre-test	76	80.4	75.7
At 5.5 min	**87.8 (*****p*** **<** **0.001)**	83.5	80.6
At 10 min	**90.2 (*****p*** **<** **0.001)**	83.1	81
PP/SBP ×100			
Pre-test	33.3%	31.1%	33.2%
At 5.5 min	**23.1% (*****p*** **<** **0.001)**	28.2%	29.5%
At 10 min	**20% (*****p*** **<** **0.001)**	27.6%	29%
Orthostatic intolerance			
OH	1/42 (2%)	0/26 (0%)	0/20 (0%)
POTS	12/42 (28%)	**12/26 (46%)**	5/20 (25%)
Presyncope/ syncope with test	7/42 (17%)	4/26 (15%)	1/20 (5%)

*Bold indicates that the Long COVID or ME/CFS group is significantly different from the Healthy Control group, with a p value as shown*.

In contrast, DBP was significantly higher at 5.5 min and at 10 min of the NLT in the Long COVID patients vs. the healthy control subjects, thereby narrowing the pulse pressure. An abnormally “narrowed” pulse pressure is defined as being one fourth or less of the systolic pressure (34, 37). The PP/SBP × 100 was significantly reduced (<25% of SBP) at 5.5 min and at 10 min of the NLT in the Long COVID patients vs. the healthy control subjects (*p* < 0.001).

**Figure 2:**
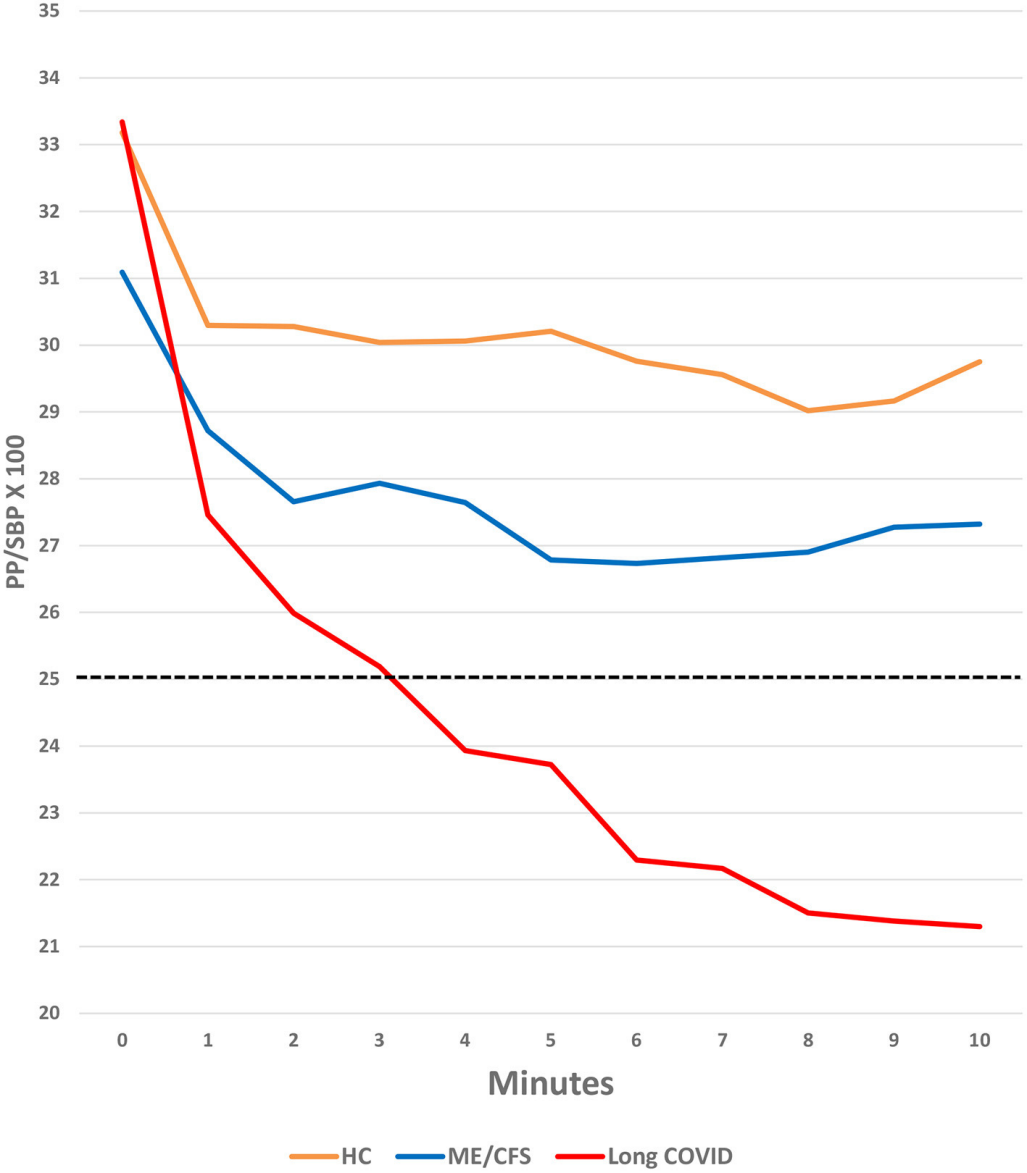
Change in PP/SBP × 100 during the NASA Lean Test in Long COVID, ME/CFS and healthy control subjects.

As shown in Figure 2, participants in all three groups had a decrease in PP/SBP × 100 in the first minute after transitioning from lying to standing. This drop stabilized at a relatively high level in the healthy control patients and stabilized later and at a lower level in the ME/CFS patients. In contrast, the PP/SBP × 100 continued to fall in the Long COVID patients, dropping to a very low 20% at 10 min (*p* < 0.001), compared to healthy control subjects.

The differences in the PP/SBP × 100 between the Long COVID and ME/CFS patients (regardless of illness duration) were not statistically significant, but the power of the study to recognize the differences as significant was inadequate. Also, the PP/SBP × 100 for the people with ME/CFS may not have been as narrow as in the people with Long COVID because, as we have shown previously (33), PP/SBP × 100 is most narrow in people with ME/CFS ill for <4 years, and then widens with increasing duration of illness. In this study, fewer than a third of the ME/CFS patients had been ill for <4 years; as in our previous study, these shorter-duration patients had lower PP/SBP × 100 values than those who had been ill longer.

### Cognitive Efficiency Testing

The effect of orthostatic challenge on the three measures of reaction time (PRT, SRT and GNG) was assessed just before, immediately after, 2 days, and 7 days after the NLT. As seen in Table 3, immediately after completion of the NLT, cognitive efficiency for all three tests of reaction time dropped in the two patient groups whereas it rose in the healthy control subjects. The difference between each of the two patient groups and the healthy control subjects was statistically significant for the PRT and SRT.

**Table 3:** Percent change from baseline to immediately post-NASA Lean Test on three tests of reaction time, in people with Long COVID, ME/CFS and healthy controls.

	**Long COVID (*N* = 42)**	**ME/CFS** **(*N* = 26)**	**Healthy controls (*N* = 20)**
Procedural reaction time	−8%***	−3%**	+6%
Simple reaction time	−4%	−8%*	+1%
Go-No-Go	–1.3%	–2%	+3%

* *Difference between Long COVID group or ME/CFS group and Healthy Controls has p-value of < 0.05.*

** *Difference between Long COVID group or ME/CFS group and Healthy Controls has p-value of < 0.01.*

*** *Difference between Long COVID group or ME/CFS group and Healthy Controls has p-value of < 0.001*.

### Functional Level

People with ME/CFS and Long COVID did no regular physical activity during leisure time (such as fitness walking or exercise classes) and stood for no more than 3–4 h per day. Healthy control subjects were physically active during leisure time two or more times per week, for a total of 30 min or more per day; none had physically-demanding occupations, such as farming, mail delivery, or stockroom worker.

## Discussion

Orthostatic intolerance and cognitive impairment are frequently experienced by people with Long COVID and people with ME/CFS. OI usually reflects dysfunction of the autonomic nervous system (ANS), and in some cases ANS dysfunction also may be the cause of cognitive impairment. Treatments are available for OI, and cognitive impairment may be one of the most common reasons for inability to function in the workplace. Moreover, persisting abnormalities of the ANS have been documented in both people with COVID-19 in the months following the acute illness (11, 23–25, 38) and in people with ME/CFS (11, 12, 26–28).

The primary purpose of our study was to evaluate the utility of simple office-based tests to primary care clinicians in providing *objective* evidence of OI and brain fog, in evaluating patients with possible Long COVID or ME/CFS. Undoubtedly, formal testing of the central and autonomic nervous system, or of cardiopulmonary exertion (such as dynamometry tilt-table testing, or ergospirography) would produce much richer information than the simple testing we evaluated. Such more rigorous testing should be conducted to better understand the underlying pathophysiology of Long COVID and ME/CFS. However, such testing is not readily available to all primary care clinicians, and can be expensive.

That may well change in the future. Major strides are being made in monitoring autonomic function using wearable devices like the Apple Watch or Fitbit device, as well as in automatically transferring electrocardiographic and heart rate variability data electronically to central databases, providing information to clinicians and investigators (39, 40). Indeed, such devices have been used to look for early warning signs of new COVID-19 infection, and it has been shown that increased heart rate variability is correlated with health benefits across many disease states, including inflammatory conditions such as COVID-19 and ME/CFS (40). However, many patients do not yet have such devices.

For these reasons, we have looked for simple, informative office-based tests to help the practicing primary care clinician assess both hemodynamic (autonomic) function and cognition. Previously, we found that two simple and inexpensive tests that can be performed in a primary care office practice produce symptomatic, hemodynamic and cognitive changes in people with ME/CFS (33), in patients without their own hemodynamic monitoring devices.

In this study, we used those simple tests to determine if the same abnormalities seen in people with ME/CFS also would be seen in people with Long COVID, particularly in light of a recent study using head-up tilt table testing indicating that they would (11).

### Symptoms

The severity of each of the symptoms was greater *pre-*NLT in the Long COVID and ME/CFS patients than in the healthy control subjects, as would be expected: the two patient groups are defined by the presence and severity of their symptoms.

The question we were addressing was whether symptoms would flare in the *post-*orthostatic challenge period. Indeed (Figure 1), there was a significant *worsening* of symptoms during and immediately after the NLT only in the two patient groups, and not in the healthy control subjects. The severity of the symptoms provoked by the NLT was slightly greater in the Long COVID group than in the ME/CFS group, but this difference in severity was not significant. The similar worsening of symptoms in both patient groups following orthostatic challenge has also been reported recently following head-up tilt table testing (11). We can only speculate as to why symptom severity remains increased seven days post-NLT, compared to pre-NLT; perhaps the response to the orthostatic challenge induces a persisting reduction in cerebral blood flow (11) or ignites low-grade neuroinflammation (41).

### Hemodynamic Changes

The most striking hemodynamic finding caused by the NLT, particularly in the people with Long COVID, was an increased diastolic blood pressure (DBP) which caused a reduced pulse pressure (PP) and an abnormally narrowed blood pressure ratio (PP/SBP × 100 < 25%), as shown in Figure 2. In people with Long COVID, the differences in DBP and PP/SBP × 100 compared to healthy control subjects were highly statistically significant (Table 2). DBP and PP/SBP × 100 values in people with ME/CFS were intermediate between values in the people with Long COVID and healthy control subjects, with lower values in PP/SBP × 100 being seen in those people with ME/CFS for <4 years, as we found in a prior study (33).

Narrow pulse pressures occur in several diseases including heart failure (decreased pump effectiveness), blood loss (decreased blood volume), aortic stenosis (reduced stroke volume), and cardiac tamponade (decreased filling time). Typically, in these cases, the narrow pulse pressure is due to a decrease in systolic pressures while diastolic pressures remain near normal. In contrast, we found that the narrow pulse pressure in our patients was due to a *rise* in DBP with fairly stable SBP, as has been reported in people with Long COVID and ME/CFS during head-up tilt table testing (11).

The elevated DBP and stable SBP we observed may reflect autonomic dysfunction–either increased sympathetic output and/or inadequate compensatory parasympathetic response. In this study we did not take measurements that would allow us to document the state of sympathetic/parasympathetic balance. Others, however, have found evidence of sympathetic/parasympathetic imbalance by measuring the nocioception level (NOL) index, a multiparameter artificial intelligence-driven index calculated from multiple physiological signals (23). Autonomic dysfunction has been documented in both ME/CFS (26–29) and in post-COVID patients (11, 23–25). The basis for dysautonomia is unclear, but autoantibodies targeting the autonomic nervous system have been demonstrated in people with ME/CFS (42–45) and autoantibodies against neural targets have been demonstrated in people following COVID-19 (46).

An alternative explanation for our findings is that the endothelial dysfunction that is well documented in COVID-19 (47) may lead to increased arteriolar tone—an imbalance between vasodilating and vasoconstricting mediators. We speculate that this may become manifest during standing because standing exaggerates the lower blood volume and lower extremity venous pooling that has been documented in ME/CFS (48, 49) and in Long COVID (50), and that vasoconstriction occurs as a compensatory mechanism.

Another alternative explanation is the hypovolemia that has been documented in both Long COVID (50) and ME/CFS (51, 52). It is normal for DBP to increase somewhat upon standing. Within 3 min of standing, gravity has created venous pooling that reduces venous return to the heart and stroke volume. This, in turn, transiently reduces blood pressure, activating arterial baroreceptors and consequent vasoconstriction, normalizing blood pressure (53). Hypovolemia should exaggerate this normal physiologic response upon standing.

We found that in people with ME/CFS, narrowed PP was seen more often in the early years of illness than later. It appears that over time, the ANS dysfunction seen in people with ME/CFS gradually changes. The physiologic mechanisms of this adaptation are unclear.

### Cognitive Changes

Three reaction time cognitive tests—assessed before, during and after the NLT—revealed that, immediately after completion of the orthostatic challenge, cognitive efficiency on all three cognitive efficiency tests dropped for both patient groups whereas it rose in the healthy control subjects, as seen in Table 3.

The slowed reaction time we observed is consistent with the subjective reports by people with Long COVID and with ME/CFS that orthostatic challenge leads to worsening in their cognitive function. Slowed reaction time is one of the most sensitive measures of impaired cognitive functioning and has been the most consistently measured objective finding in ME/CFS neurocognitive research (12).

### Underlying Brain Pathophysiology

The pathophysiology of slowed reaction time is uncertain. Many different abnormalities of the central and autonomic nervous system have been identified in people with ME/CFS, as summarized in detail elsewhere (54). When cognitive impairment is worsened by orthostatic challenge, as in this study, we think a likely contributing factor is a generalized reduction in brain perfusion. In 1995, a specific pattern of hypoperfusion of the brainstem was detected in people with ME/CFS, but not in patients with major depression, epilepsy or healthy controls (55). Since then, several studies have found that many parts of the brain in people with ME/CFS have reduced blood flow (56–60). Furthermore, reduced regional cerebral blood flow is associated with increased symptom severity in people with ME/CFS (61). Recently, cerebral hypoperfusion also has been documented in most people with Long COVID (11, 25).

In people with ME/CFS, dramatic reductions in cerebral perfusion are often triggered by head-up tilt table testing (59), and even by mild orthostatic stress (60, 62), and this reduction in perfusion can persist following completion of a head-up tilt table test (63)The reductions in cerebral perfusion are not explained by a drop in blood pressure (59).

Chronic cerebral hypoperfusion is associated with neurocognitive disorders and cognitive impairment (64) and it is plausible that chronic intermittent brain hypoperfusion in ME/CFS caused the cognitive impairment demonstrated in this study. It is notable that cognitive efficiency in ME/CFS patients did not improve above baseline for the three reaction time tests even 7 days after the NLT. While cognitive efficiency worsened for Long COVID patients immediately after the NLT, SRT and GNG, cognitive efficiency ultimately improved to a level similar to that in healthy controls. Chronic intermittent hypoperfusion also may be contributing to the neuroinflammation (41), oxidative stress (65, 66), and altered brain connectivity (67) that are seen in people with ME/CFS.

### Underlying Systemic Pathophysiology

In addition to the central nervous system pathology described above, several systemic pathophysiological abnormalities also have been described in people with COVID-19 and with ME/CFS when they are *at rest:* a hypometabolic state, abnormalities of energy metabolism, activation of some parts of the innate immune system, production of autoantibodies and redox imbalance (54, 66). In the study we report here, we sought to determine if any of these abnormalities might be aggravated by the orthostatic challenge that causes worsened symptoms. The rationale for doing so is that while some of the underlying physiological abnormalities in these illnesses may well be epiphenomena, other abnormalities—such as those that worsen when symptoms worsen following provocation by a stressor—are more likely to be central to the pathogenesis of those symptoms. Analysis of these data are underway and will be the subject of future reports.

## Conclusions

Some people with acute COVID-19 are left with persistent, debilitating fatigue, cognitive impairment, orthostatic intolerance (OI) and other symptoms (“Long COVID”), a condition similar to ME/CFS and other post-infectious fatigue syndromes. This study finds that simple tests of orthostatic stress and cognition in an office practice setting can elicit worsened symptoms, impaired cognition and characteristic hemodynamic changes (mainly narrowed pulse pressure) in people with Long COVID and ME/CFS. This provides objective confirmation of the orthostatic intolerance and brain fog reported by both patient groups. It also underscores the need to detect orthostatic intolerance early because early treatment theoretically could mitigate progression to chronic and less reversible conditions and cognitive impairment.

## Data Availability Statement

The raw data supporting the conclusions of this article will be made available by the authors, without undue reservation.

## Ethics Statement

The studies involving human participants were reviewed and approved by Columbia University Institutional Review Board (protocol #AAAR9065). The patients/participants provided their written informed consent to participate in this study.

## Author Contributions

SV, LB, WL, and AK conceptualized the study design and supervised the data collection. SF and GS were responsible for statistical analysis. SH, KS, JB, and SA were responsible for patient recruitment and clinical assessment. SV, LB, GS, WL, and AK were responsible for writing the manuscript. All authors contributed to the article and approved the submitted version.

## Funding

The study was supported by a grant from the National Institutes of Health to Columbia University (AI138370) and by several private donations.

## Conflict of Interest

The authors declare that the research was conducted in the absence of any commercial or financial relationships that could be construed as a potential conflict of interest.

## Publisher's Note

All claims expressed in this article are solely those of the authors and do not necessarily represent those of their affiliated organizations, or those of the publisher, the editors and the reviewers. Any product that may be evaluated in this article, or claim that may be made by its manufacturer, is not guaranteed or endorsed by the publisher.
